# Role of *SOX9* in the Etiology of Pierre-Robin Syndrome

**Published:** 2013-05

**Authors:** Selvi R, Mukunda Priyanka A

**Affiliations:** 1 Department of Human Genetics, Sri Ramachandra University, Porur, Chennai-116, Tamil Nadu, India

**Keywords:** Cleft lip and Palate, Cytogenetics, PCR, Pierre Robin Syndrome *SOX9*

## Abstract

***Objective(s*** ***:*** Cleft lip/palate are common congenital anomalies, affecting approximately 2/1000 live births. Pierre Robin Sequence is a subgroup of the cleft palate population. Chromosomal abnormalities near the SOX9 gene disrupt the regulation of this gene and prevent the SOX9 protein from properly controlling the development of facial structures, which leads to isolated PRS. The present study was conducted to identify the role of the *SOX9* gene in the etiology of Pierre robin syndrome and to study the association of *SOX9* and PRS in regulating morphogenesis of the face in individuals with Cleft lip/Palate using the PCR technique and GTG banding.

***Materials and Methods:*** Molecular and cytogenetic analysis was performed in 27 subjects with cleft lip/palate and 13 age matched controls. DNA was isolated and PCR was performed for the amplification of the gene of interest and the products were run on a 2% Agarose gel and the band patterns were analyzed. The chromosomal abnormalities were analyzed from the cultured lymphocytes after GTG banding.

***Result:*** Out of 27 patients screened, deletion of the *SOX9* gene was observed in 1 case for exon1 and in 2 cases for exon2. The cytogenetic analysis showed no structural or numerical abnormalities and all the patients showed normal karyotype.

***Conclusion:*** The results of molecular methods showed a positive association suggesting that the SOX9 gene is of particular importance, but the cytogenetic study didn’t seem to show a stronger association suggesting that, this method would not identify disease genes acting via other mechanisms of genetic dominance and also due to the fact that Cleft lip / palate has a multifactorial inheritance.

## Introduction

Oral clefts are the most common birth defects, affecting approximately 2/1000 newborns worldwide (1, 2). Depending on geographic origin, racial and ethnic backgrounds it can be associated with other anomalies**.** Cleft lip and palate (CL/P) are a diverse and variable congenital anomaly, while over 300 distinct syndromes associated with oral clefts have been identified. Cleft palate is a common birth defect, but its etiopathogenesis is mostly unknown. Several studies have shown that cleft palate have a strong genetic component.

 A cleft lip is a split in the upper lip, which can be unilateral or bilateral, creating a wider opening into the nose. A cleft palate is a split in the roof of the mouth. This leaves a hole between the nose and the mouth. Sometimes a cleft lip and cleft palate occur together in the same individual. Cleft lip deformity is established in the first 6 weeks of life, possibly due to the failure of fusion of maxillary and medial nasal processes or may be due to incomplete mesoderm ingrowths into the processes. The extent of deficiency determines the extent of the cleft (3). 

Pierre Robin sequence (PRS) is one of the most common syndromes in patients with CL/P worldwide; it is characterized by three features, micrognathia, glossoptosis and cleft palate, due to underdevelopment of the lower jaw. The Pierre Robin sequence is a clinically well-defined subgroup of the CL/P population with an unknown aetiology. The overall incidence of PRS is 1 in 8500 to 14,000 births in the general population, which is same for both sexes (4).

Robin sequence is a condition with multiple causes. Most cases are thought to result from hypoplasia of the mandible that occurs before the ninth week of development. Pierre Robin Sequence is called a sequence and not a syndrome because the underdeveloped lower jaw begins a sequence of events during embryology, which leads to the abnormal displacement of the tongue and subsequent formation of a cleft palate. It is also known as the Pierre Robin malformation, a congenital condition of facial abnormalities in humans (5). PRS is a sequence with a chain of certain developmental malformations, one entailing the next. Earlier studies on genetics of Pierre Robin sequence revealed deletions; duplications; translocations; and mutations in chromosomes 1 to 6, 10 to 13, and 16 to 18. Regions in chromosome 2 (2q24.1-33.3), chromosome 4 (4q32-qter), chromosome 11 (11q21-q23.1), and chromosome 17 (17q21-q24.3) were mostly reported. Several lines of evidence for the existence of a 17q24 locus underlying PRS, including linkage analysis results showed a clustering of translocation breakpoints and micro deletions (6).

Genetic factors contributing to a cleft lip and palate formation have been identified for some syndromic cases, but knowledge about genetic factors that contribute to the more common isolated cases of cleft lip/palate is still ambiguous. Genetic causes of clefting also include chromosomal rearrangements, genetic susceptibility to teratogenic exposures, and complex genetic contributions of multiple genes. Cleft lip and palate also occur as part of over 100 syndromes and some of the most common conditions include Pierre Robin Syndrome, Stickler Syndrome, Down's syndrome, Treacher Collins' Syndrome, Van der Woude syndrome, 22q11.2 deletion syndrome, trisomy 13, and trisomy 18 (7).

Previous studies reveal that PRS not only cause dysregulation of the *SOX9* gene but also cause dysregulation of the KCNJ2 gene, which plays a significant role in Stickler Syndrome. Some cases of Robin sequence may thus result from developmental misexpression of *SOX9* due to disruption of very-long-range-cis regulatory elements. Mutation in genes such as GAD67, PVRL1, COL11A1 and COL11A2, have also been noted in children with PRS. However, up to 25% of children with PRS will have a family member with a cleft palate, which is a more common association than might be expected if there was no genetic role for this problem (8).

The official name of the *SOX9* gene is “SRY (sex determining region Y)-box 9”. The *SOX9* gene provides instructions for making a protein that plays a critical role during embryonic development. The SOX9 protein is especially important for the development of the skeleton and reproductive system. This protein binds to specific regions of DNA and regulates the activity of other genes. On the basis of this action, the SOX9 protein is called a transcription factor. 

The *SOX9* gene is located on the long (q) arm of chromosome 17 at position 23 (17q23). More precisely, the *SOX9* gene is located from base pair 70,117,160 to base pair 70,122,560 on chromosome 17. The mutations in *SOX9* prevent the production of the SOX9 protein or result in a protein with impaired ability to function as a transcription factor. About 5 percent of cases are caused by chromosomal abnormalities that occur around the *SOX9* gene. These chromosomal abnormalities disrupt regions of DNA that normally regulate the activity of the *SOX9* gene. All of these genetic changes prevent the SOX9 protein from properly controlling the genes essential for normal development of the skeleton, reproductive system, and other parts of the body (9). 

It is well known that PRS is a heterogeneous group and has a partly genetic aetiology; because it remains a part of many Mendelian syndromes, the aetiology of isolated PRS is generally unknown. In general, the gene identification process for orofacial clefting is still in the early stages due to the genetic complexity of clefting. Therefore, it is anticipated that there are additional genes involved in orofacial clefting that have yet to be identified and the functional effects of identified mutations have yet to be discerned. The identification of a disrupted gene(s) will enable further genetic studies that may elucidate the underlying aetiology of PRS, leading to a better understanding of craniofacial development. 

The main aim of this study was to identify the role of the *SOX9* gene in the etiology of Pierre Robin syndrome and to study the association between *SOX9* and PRS in regulating morphogenesis of the face in individuals with Cleft lip/Palate using the PCR technique and GTG banding. 

## Materials and Methods

Ethical clearance was obtained from the Committee for Students Proposals at the Sri Ramachandra University, in order to proceed with the sample collection. A formalized consent form was used to procure approval from the subjects. They were given complete information about the study. Demographic and family information were obtained from the subjects and respective guardian(s).

3 ml of Peripheral blood samples were collected in EDTA Vacutainers and 2 ml in heparinised vacutainers by vein puncture from 27 Patients with Cleft lip/Palate who were attending the Department of Plastic & Reconstructive surgery at the Sri Ramachandra University and 13 age – matched controls were enrolled in this study. Subjects of both sexes and age groups between 6-25yrs were taken into consideration. 

Heparinised blood samples were used for chromosomal analysis, where the cells were cultured for 72 hr at 37°C with 5% CO_2 _and were arrested at the metaphase stage and the pellet was then casted onto a clean glass slide and the slides were aged overnight at 60°C and banded with trypsin and giemsa and analyzed under the microscope. 25 metaphases were analyzed under 100X magnification and karyotyped using the cytovision software to rule out the chromosomal abnormalities. 

The EDTA samples were used for the molecular study. The DNA was isolated by the salting out method (10). The leucocytes were separated from the red blood cells by the addition of red cell lysis buffer followed by centrifugation to pellet down the intact leucocytes. DNA containing leucocytes were lysed using nucleated lysis buffer and 10% SDS and incubated at 55°C for 20 min - 2 hr to degrade integral proteins and thereby release the DNA. The lysate was transferred to an eppendorf tube and 400 µl of 5M NaCl was added to the lysate and centrifuged at 10000rpm for 15 min at 4°C. To the supernatant, double the volume of ethanol was added and the tube was gently inverted until the DNA precipitated out. The DNA was transferred to an eppendorf tube and 500 µl of 70% ethanol was added, centrifuged at 2000 g for 5 min at 4°C. The supernatant was discarded and the pellet was air-dried. To the pellet 150 µl of TE buffer was added and the solution was stored in the refrigerator at 4°C. The quality and the quantity of the DNA were checked using 0.8% agarose and Nanodrop. 

The isolated DNA was then subjected to PCR (11). A gradient PCR was performed to identify the annealing temperatures for both exon1 and 2 followed by a standard PCR. The primer sequence for exon 1 and 2 were as follows; forward 5’GCTTCTCGCCTTTCCCGGCC3’ reverse 5’ GGGGCAAATCAGCCCTGACCAG3’ forward 5’CGACCTGACAGTTTGGCGGAT3’ reverse 5’ GGTGTGCCAGGCGGGACG 3’. All the reagents were briefly centrifuged in a Spinwin microcentrifuge after thawing. A master mix was prepared using nuclease free water, 10X PCR buffer, dNTPs, Primers, Taq DNA Polymerase and template DNA and it was then aliquoted into individual PCR tubes. The tubes were placed in the thermocycler and the conditions were maintained as follows; initial denaturation at 95°C for 5 min, denaturation at 94°C for 40 sec, annealing at 64 °C for 30 sec for exon 1 and 58°C for 30 sec for exon 2, extension at 72°C for 1 min 10 sec, final extension at 72°C for 5 min and on completion, the PCR products were run on a 2% agarose gel and the gel was then visualized using a gel documentation system in order to check for the amplification of the gene of interest. 

## Results

The DNA was isolated from the peripheral blood sample and the DNA quality on average was found to be between 1.6 and 1.9. The annealing temperature was identified by gradient PCR as 64^o^C and 58^o^C respectively for exon 1 and exon 2 of the *SOX9* gene. Followed by the standard PCR, the products were run on a 2% agarose gel and the band patterns were visualized using the gel documentation system. For exon1 deletion of the SOX9 gene was observed in 1 case and for exon2 the same was observed in 2 cases. Out of 27 patients screened for exon1, in 26 patients a band was observed at the 553 base pair and for exon 2, in 25 patients a band was observed at the 384 base pair, corresponding to the bands as seen in control samples. The cytogenetic analysis showed no structural or numerical abnormalities and all the patients showed a normal karyotype.

**Figure 1:**
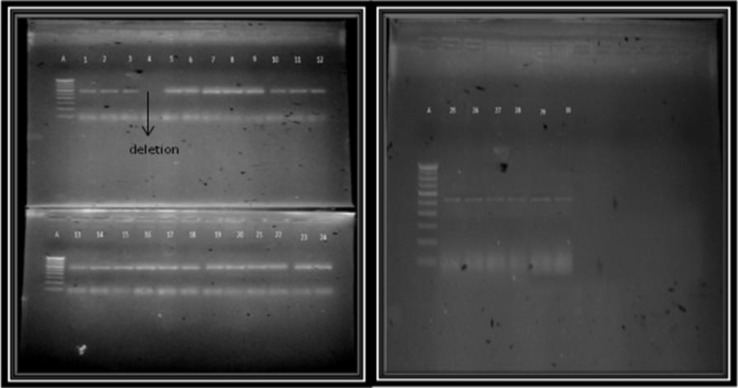
Agarose gel electrophoresis showing band patterns (Exon 1)

A=100 BASE PAIRS DNA LADDER

Lane A – DNA ladder

Lane 2-12 – Patient’s sample

Lane 14-24, 26-30 – Patient’s sample

Lane 1,13&25 – Normal control

**Figure 2:**
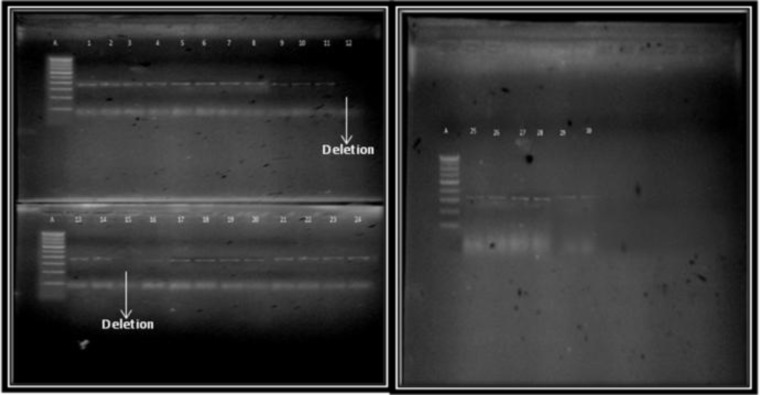
Agarose gel electrophoresis showing band patterns (Exon 2)

A=100 BASE PAIRS DNA LADDER

Lane A – DNA ladder

Lane 2-12 – Patient’s sample

Lane 14-24, 26-30– Patient’s sample

Lane 1,13&25 – Normal control

**Figure 3:**
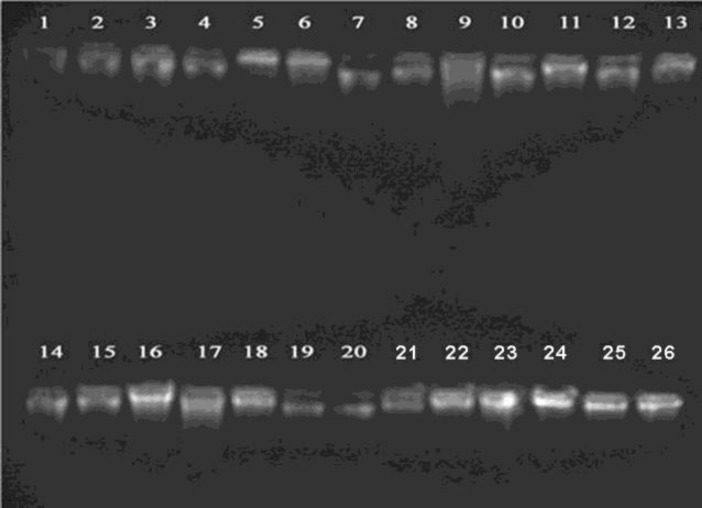
DNA Quality Check on 0.8% Agarose gel

Lane 1-26 – Patient’s DNA sample

Lane 4- DNA sample of 4^th^ patient

Lane 12- DNA sample of 12^th^ patient

Lane 15- DNA sample of 15^th^ patient

## Discussion

Pierre Robin Sequence is a subgroup of the cleft lip/Palate population with an unknown aetiology. Robin sequence is etiologically heterogenous. Etiologic heterogeneity suggests pathogenetic heterogeneity and phenotypic variability. Jakobsen *et al* compared data of several databases and proposed genes that might participate in the etiology of Robin sequence, which included *GAD67* on 2q31, *PVRL1* on 11q23-q24 and *SOX9* on 17q24.3-q25.1. Melkoniemi *et al* detected disease-associated mutations in *COL11A1* and *COL11A2* genes in some patients with non-syndromic Robin sequence (12). Besides GAD67 and PVRL1, the SOX9 transcription factor was found to play an important role in regulating morphogenesis of the face in individuals with Cleft lip/Palate. SOX9 has been shown to regulate collagen expression during cartilage and endochondral bone formation (Bell *et al*, 1997; Mori-Akiyama *et al*, 2003) (13).

Mutations in genes coding for collagen types have been found in patients with PRS (Melkoniemi *et al*, 2003), indicating a regulatory pathway involving SOX9 and genes coding for collagen, whose disruption can cause PRS (14). Furthermore, Velagaleti *et al* (2005) suggested that PRS might result from dysregulation of SOX9 (15). It has been known that the Sox9 is a member of the Sox family of transcription factors and regulates both chondrogenesis and sex determination. Mutations in the SOX9 gene cause skeletal abnormalities that often include the cleft palate. This led us to consider SOX9 as the primary candidate gene in patients with cleft lip/ palate associated with Pierre Robin syndrome.

In the present study we acquired the genetic information of PRS from previous literature and from cytogenetic database to facilitate focused genetic studies of PRS. The general aim of the study was to identify the role of SOX9 in the aetiology of Pierre Robin syndrome associated with cleft lip / palate, which is a common and complex congenital malformation caused by environmental and genetic factors. This was achieved by studying in detail the Pierre Robin Sequence (PRS), which is a subgroup of the cleft lip and palate population. Moreover, in the present study Polymerase Chain Reaction (PCR) and chromosomal analysis by GTG banding technique using peripheral blood lymphocytes were performed to identify the mutation and abnormalities in chromosomes. 

Results of the molecular study were promising which showed deletion of *SOX9* in exon1 in one patient and in exon 2 in 2 patients, suggesting that the SOX9 gene is of particular importance, but the cytogenetic study didn’t seem to be strongly in favour of the location of the gene responsible for PRS at chromosome 17q suggesting that, this method would not identify disease genes acting via other mechanisms of genetic dominance and also due to the fact that Cleft lip / palate has a multifactorial inheritance. Even though karyotyping is limited by efficiency in the detection of subtle chromosomal alteration (16) it still serves as the basis for identifying gross chromosomal aberrations. To identify the presence of subtle chromosomal alterations in complex disorders like the cleft lip and palate, karyotyping has to be combined with techniques like Fluorescent in situ hybridization. Furthermore, mutation analyses of the genes in high risk families may provide us with information on the genetic contribution of these genes in the general CL/P population. A major distinction should be made between isolated occurrences of Robin sequence and cases in which Robin sequence is part of a recognized syndrome, or a part of a complex of multiple anomalies, or part of an unrecognized syndrome. Though there are many genes involved in the formation of the lip and palate during embryogenesis, the environmental factors teratogens and nutrition also have an influence on the developing fetus, which should also be considered. 

## Conclusion

The study results from molecular methods showed the association between *SOX9* gene and Pierre Robin syndrome suggesting that SOX9 gene is of particular importance. To conclude, efforts should be focused on the genomic regions and genes, which are more commonly associated with PRS. Further molecular cytogenetic characterization of SOX9 region should be done to identify the putative gene for isolated PRS. To determine the extent of the involvement of SOX9 in PRS, sequencing of the SOX9 candidate cis-regulatory regions and signaling partners in a larger group of patients are needed. 
